# Quarterly Summary

**Published:** 1857-01

**Authors:** 


### QUARTERLY SUMMARY.

1.—*Ammonia in Blood.*—Dr. Richardson has recently taken the Astley Cooper prize for an essay proving that ammonia is a normal constituent of the blood, and that it keeps it in a fluid state. He demonstrates its existence in the air expelled from the lungs during expiration by a very simple experiment. A glass plate is covered by a few drops of perfectly pure muriatic acid. This is breathed upon for a number of times, and the fluid still on the surface of glass is evaporated at a gentle heat. Crystals of chloride of ammonium can then be recognized by the microscope. The same result can be attained by allowing the halitus from fresh blood in a vessel covered likewise by a plate moistened with muriatic acid. If chloride of platinum be substituted for the acid, beautiful gold-colored crystals of the double chloride of ammonia and platinum are obtained.

The quantity of ammonia in the breath of different persons varies with the state of health and other circumstances of the individual experimented on. During the act of digestion the quantity is less than when fasting.

2.—*Functions of the Cerebellum.*—Dr. Andrews has published in the Peninsular Journal of Medicine a paper on this subject, in which he takes the ground that the control over the muscular actions of the different limbs, resides in different lobes of this organ. He thinks that the middle lobe co-ordinates the muscular movements of the upper or anterior extremities and that the lateral lobes preside over the lower or posterior extremities. He derives his arguments from comparative anatomy, and shows that in those animals in which the greatest muscular power resides in the anterior extremities, the middle lobe preponderates, while in those which use the posterior extremities most, the lateral lobes are largest.

3.—*Chemistry of Respiration.*—In the British and Foreign Medico-Chirurgical Review for October, Mr. Harley has an article on this subject, in which he recounts some experiments instituted to refute the notion of Magnus that the gases, interchanged in this great function, was simply in the state of mechanical admixture with the blood, and to determine the component with which they were combined.

He employed a retort partly filled with blood, which had previously been made to take up as much atmospheric air as it would absorb, and consequently had lost all the other gases which could be displaced by physical means. After this, the blood being allowed to stand for some time at a moderate temperature, the escaping gases were collected in a graduated receiver, analyzed and compared with the constitution of the atmosphere. The oxygen was found to have notably diminished, and the carbonic acid to have increased, but not in a ratio to the disappearance of oxygen, more of the latter gas having been lost than was contained in the carbonic acid. Hence not only has it been consumed to form carbonic acid, but it has also been combined with some of the organic matters which have retained it. Some of it may have combined with hydrogen to form water, but this the experimenter did not attempt to ascertain. He supposes that a portion of this retained matter may have combined with some of the constituents of the blood, adapt them for assimilation, and that another portion may have united with the effete matters to make them more suitable for elimination. This process requires a certain amount of time, a fact shown by an experiment which demonstrated that, when the air was merely passed through the blood, a very slight change took place, whereas when it was allowed to remain for some time, that alteration was well marked.

The operator also instituted experiments to determine what constituents of the blood possessed this power of so combining with oxygen. He discovered that albumen, fibrin and hæmatin all give results similar to those obtained from the mixed fluid.

The results of these experiments are thus summed up by the author.

"Firstly—That blood has the property of chemically combining with the respired oxygen.

Secondly—That the coagulum, the serima, fibrin, albumen and hæmatin are among the substances possessing this property.

Thirdly—That these substances not only become oxydized, but also yield carbonic acid gas.

Fourthly—That time, temperature, and the presence of foreign matter, are among the agents which modify these changes."

4.—*Anæsthetics.*—Dr. J. Henry Clark, of Newark, New Jersey, publishes in the September number of the New York Journal of Medicine, a paper on the constitutional effects of anæsthetic agents. He believes that protracted ill consequences follow their administration, and relates three cases occurring in his own practice, in which habitual costiveness and indigestion, mental feebleness, and neuralgic pains, continued for a space of time varying from a few months to two years. He also quotes other physicians to the effect that permanent idiocy and mania have followed the use of these powerful agents. The question is an important one, and we hope to see it fully and fairly discussed.

### CORRESPONDENCE.

